# Development of a post-treatment prognostic model for hepatocellular carcinoma based on nutritional, immune, and inflammatory scoring systems and REDCap-enabled follow-up

**DOI:** 10.3389/fonc.2026.1683412

**Published:** 2026-03-05

**Authors:** Xuemei Liu, Chunxiao Wei, Maoyu Jiang, Fengqiao Huang, Haiyan Wu, Xueyin Liao, Zhong Huang, Zhenyu Liu

**Affiliations:** Department of Oncology, Kaiyuan Langdong Hospital of Guangxi Medical University, Nanning, China

**Keywords:** follow-up cohort, hepatocellular carcinoma, inflammation score, prognostic model, REDCap

## Abstract

**Background:**

This study examined the association between pre-treatment inflammation, immune cell- and nutrition/metabolism-related scores, and prognosis of patients with hepatocellular carcinoma (HCC) post-treatment.

**Methods:**

This study collected clinical data on demographics, pretreatment blood tests, pathology, and follow-up. Key markers included C-reactive protein, albumin, neutrophil and lymphocyte counts, creatinine, bilirubin, international normalized ratio, tumor size and number, alpha-fetoprotein, platelet count, and CD4+/CD8+ T-cell levels. Disease-free survival (DFS) was calculated from treatment to recurrence. Twelve scores were derived. Kaplan–Meier and univariate Cox analyses identified significant predictors, followed by multivariate Cox models to determine independent risk factors. Logistic regression and receiver operating characteristic (ROC) analyses assessed predictive performance. Scores were grouped as inflammation-, metabolism-, or immune-related to construct nomograms and evaluate C-index values using R software.

**Results:**

Except for Gender (*p* = 0.019), all other clinical characteristics showed no statistically significant differences between the training and validation sets (*p* > 0.05).Univariate Cox regression showed that pre-albumin (P = 0.01), PNI (P < 0.001), TBS (P = 0.01), ALBI (P < 0.001), PALBI (P < 0.001), and CRAFITY (P < 0.001) were significantly associated with DFS. Multivariate analysis identified PALBI (P = 0.03) and CRAFITY (P = 0.04) as independent predictors. A prognostic model was constructed: Risk score = 0.03903 × TBS + 0.79809 × PALBI + 0.40881 × CRAFITY, stratifying patients into high- and low-risk groups. Kaplan–Meier analysis showed significantly better DFS in the low-risk group (P = 0.001). ROC analysis for 1- and 2-year DFS yielded AUCs of 0.69 and 0.75. Logistic regression confirmed the risk score as a predictor of mortality (P = 0.002, AUC = 0.644). Excluding TBS, the remaining scores were grouped into inflammation-related, nutrition/metabolism-related, and immune-related categories. Corresponding nomograms showed good calibration, with C-index values of 0.610, 0.581, and 0.575, respectively.

**Conclusion:**

Pre-treatment PALBI and CRAFITY scores are independent prognostic factors for post-treatment survival among patients with HCC, with inflammation-related scores providing superior predictive value for DFS compared to metabolism- and immune-related scores.

## 1 Introduction

Hepatocellular carcinoma (HCC) ranks among the most common malignant tumors globally, with patients frequently diagnosed at an advanced stage, leading to a poor prognosis (1). Its onset is closely associated with uncontrolled hepatocyte proliferation and a deteriorating liver regeneration microenvironment (2). A previous study has demonstrated that men are more predisposed to developing HCC than women, with a male-to-female ratio reaching 3.7:1 in high-incidence regions (3), and the average age at diagnosis ranges between 50 and 60 years (4). The occurrence of HCC is often accompanied by chronic liver inflammation and fibrosis, involving multiple risk factors, including hepatitis B virus (HBV) and/or hepatitis C virus (HCV) infection, aflatoxin exposure, alcohol abuse, metabolic syndrome, obesity, and diabetes, all of which are associated with environmental and genetic predisposition to HCC (5).

Recent studies have demonstrated significant advances in understanding the molecular and immunological characteristics of HCC. One integrative multi-omics analysis revealed that abnormalities in circadian rhythm–related genes can classify HCC into three distinct molecular subtypes, which differ markedly in molecular features, tumor grade, and prognosis, suggesting that circadian rhythm disruption plays a critical role in HCC progression (6). In terms of multi-omics approaches, another study proposed a noninvasive MRI-based radiomic model capable of evaluating the tumor immune microenvironment and predicting both prognosis and response to immunotherapy. This Radiomic Immunoscore showed high AUC values in the validation cohort, indicating strong potential for use in individualized treatment. Together, these studies highlight the importance of molecular subtyping and immune profiling in advancing precision diagnosis and personalized therapy for HCC (7).

Surgery remains the first-line and only potentially curative treatment for HCC, providing the optimal opportunity for long-term survival. However, due to the high proportion of advanced-stage diagnoses, surgical eligibility is limited. Advancements in targeted immunotherapy and radiotherapy have facilitated the investigation of multimodal conversion therapies aimed at reducing the proportion of unresectable cases and minimizing post-treatment recurrence, thereby enhancing long-term outcomes for patients with advanced HCC (8). However, due to the insidious nature of HCC, high recurrence rates after resection, and frequent failure of liver transplantation, treatment still faces numerous challenges (9). Tumor recurrence, which is a major factor affecting patient prognosis, is one of the most common complications following surgical resection of HCC. Preventing recurrence has emerged as a substantial clinical challenge in HCC treatment (10). Most studies demonstrated that the peak period for recurrence occurs within 1–2 years post-surgery, and patients with early recurrence (defined as recurrence within 2 years) generally have a poorer prognosis than those with late recurrence (11). Therefore, early intervention for patients at high risk of recurrence may enhance post-treatment survival (12). The time interval between surgical resection and recurrence—referred to as time to recurrence—is frequently utilized to assess HCC recurrence (13). Increasing evidence indicates that inflammation is closely related to the malignant behavior of HCC and substantially affects its prognosis (14). HCC typically develops in the context of chronic hepatitis or liver injury, where inflammation and the tumor microenvironment are essential in its onset, progression, and metastasis through various molecular mechanisms (15). Moreover, inflammation-based markers have exhibited promise in predicting the prognosis of patients with HCC, offering new insights for preventing post-treatment recurrence (16).

Pre-treatment inflammatory indicators and multidimensional scoring systems offer precise prognostic classification for patients with HCC by quantifying systemic inflammation, liver functional reserve, immune status, and tumor burden. The modified Glasgow Prognostic Score (mGPS), based on C-reactive protein (CRP) and albumin (ALB) levels, evaluates systemic inflammation and nutritional status; a higher score signifies a poorer prognosis (17, 18). Mechanistically, elevated CRP indicates activated pro-inflammatory and immunosuppressive responses, whereas low ALB indicates malnutrition and impaired hepatic synthetic function (18). Studies have demonstrated that mGPS is an independent predictor of overall survival (OS) and disease-free survival (DFS) among patients with HCC, demonstrating strong prognostic value in resectable cases (hazard ratio [HR] = 1.8–2.3). Meta-analyses have confirmed its significant association with OS (*P* < 0.001) (17, 18). The prognostic nutritional index (PNI), calculated based on serum ALB levels and lymphocyte count, evaluates the nutritional and immune status of patients with HCC, with a low PNI indicating impaired immune function (19, 20). Mechanistically, low ALB indicates malnutrition, but reduced lymphocyte levels impair antitumor immunity. Studies have demonstrated that PNI is an independent predictor of OS among patients with HCC (HR = 1.45, *P* < 0.001), with superior prognostic value compared to alpha-fetoprotein (AFP) and portal vein thrombosis in advanced cases (19). A meta-analysis of eight studies confirmed a significant association between low PNI and 1-, 3-, and 5-year OS (odds ratio [OR] = 2.1, 95% confidence interval [CI]: 1.6–2.8) (20). Among patients receiving immunotherapy, those with PNI ≥ 45 exhibited a median OS of 23.9 months, markedly higher than the 11.7 months observed in patients with PNI < 45 (21). The platelet-albumin-bilirubin (PALBI) score, an extension of the ALB-bilirubin (ALBI) score including platelet count, offers a comprehensive assessment of liver function and portal hypertension (22). It serves as an independent risk factor for post-treatment recurrence among patients with AFP-negative HCC (HR = 1.73), with a combined model including microvascular invasion (MVI) and tumor size attaining a C-index of 0.704 (22). PALBI exhibits enhanced predictive accuracy for HBV-related HCC recurrence (area under the curve [AUC] = 0.78) compared to ALBI (0.71) and Child-Pugh (0.69) (23). Furthermore, a post-treatment increase in PALBI (ΔPALBI > 0) is associated with a 33% higher risk of recurrence (*P* = 0.042) (24). The model for end-stage liver disease (MELD) score, which incorporates bilirubin, creatinine, and international normalized ratio (INR), evaluates liver and kidney function and coagulation status. It accurately predicts post-treatment liver failure, with patients with cirrhosis exhibiting a MELD score > 9 facing a fivefold increased risk (AUC = 0.83) (25). Furthermore, MELD offers survival stratification, with each 10-point increase associated with a 25%–60% increase in 3-month mortality, peaking at 71.3% when MELD ≥ 40 (26). The age-male-ALBI-platelet score (aMAP), which combines age, sex, ALBI, and platelet count to evaluate a patient’s baseline condition and recurrence risk. It has strong prognostic value, with patients scoring > 60 exhibiting markedly shorter recurrence-free survival following curative treatment, especially among those with virus-related HCC (*P* < 0.001) (27). In patients undergoing radiofrequency ablation, an aMAP score > 67.8 is associated with a 5.3-fold higher risk of late recurrence (HR = 5.3, *P* < 0.001) (28). Furthermore, among patients with chronic hepatitis B, the 10-year HCC incidence in the high-risk group (aMAP ≥ 60) reaches up to 30.01% (29). CRP and AFP in immunotherapy (CRAFITY) score, which combines AFP and CRP levels, indicates tumor aggressiveness and the immune microenvironment (30). A low score (0 points) is associated with markedly improved OS and a higher objective response rate (36.4%) among patients undergoing immunotherapy (*P* = 0.044) (30), while meta-analyses revealed strong correlations between low CRAFITY and improved OS (HR = 0.22) and progression-free survival (PFS, HR = 0.36) (31); a score of 2 predicts higher risk of grade ≥ 3 liver injury (*P* < 0.01) (32). Systemic inflammation indicators—platelet-lymphocyte ratio (PLR), neutrophil-lymphocyte ratio (NLR), and systemic immune-inflammation index (SII)—indicate inflammation and impaired immune surveillance (33, 34); among them, SII ≥ 330 is associated with poor OS (HR = 1.8) and increased circulating tumor cells (*P* = 0.029), with greater predictive sensitivity than PLR/NLR (AUC = 0.69 versus 0.61) (33, 35). Furthermore, CD4/CD8 ratio serves as an immune status marker, where a low ratio (< 1.0) indicates T cell exhaustion and immunosenescence (HR = 1.4), and CD8+ T cell infiltration is significantly correlated with OS in HCC (*P* = 0.07) (36–38).

This study utilized follow-up data and incorporated multiple pre-treatment inflammatory and multidimensional scoring systems (ALBI, PALBI, mGPS, PNI, and CRAFITY) to comprehensively evaluate liver functional reserve, systemic inflammation, immunonutritional status, and tumor biological behavior to enhance the accuracy of post-treatment prognostic stratification in patients with HCC (17, 23, 39, 40). The findings revealed that combined models, including ALBI-PNI, exhibited superior prognostic efficacy compared to conventional BCLC staging (OS C-index up to 0.69) (41), and inflammation-nutrition scores such as CAR/mGPS exhibit promising predictive value for OS across various cancer types (42). Although certain single indicators may have limited predictive value in specific cancers (43), their combined application can mitigate these limitations, offering robust support for pre-treatment screening of high-risk recurrence, assessing eligibility for immunotherapy, and informing individualized treatment strategies. This study emphasizes the strong association between pre-treatment inflammatory response, immunonutritional status, liver function, and post-treatment prognosis in liver cancer, highlighting the potential of multi-indicator combined modeling to enhance the accuracy of recurrence risk prediction and optimize personalized therapeutic strategies, with significant clinical relevance and application prospects.

## 2 Materials and methods

### 2.1 Clinically enrolled patients

**Figure 1:**
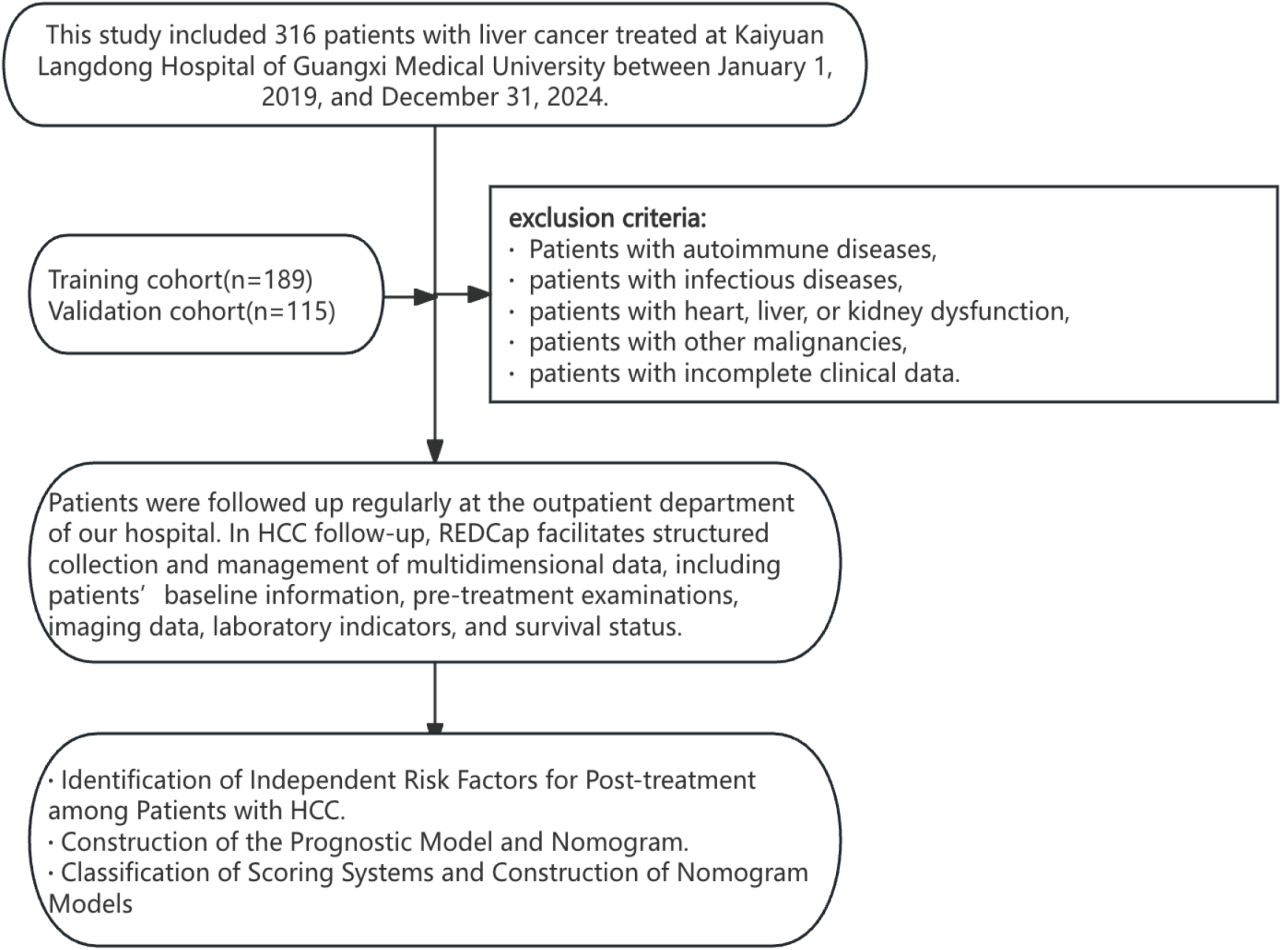
The workflow of this article.

This study included 316 patients with liver cancer treated at Kaiyuan Langdong Hospital of Guangxi Medical University between January 1, 2019, and December 31, 2024. After screening, 304 patients met the inclusion criteria and were enrolled in the study. We randomly assigned 189 and 115 patients who met the inclusion and exclusion criteria to the training and validation cohorts, respectively. Inclusion criteria were as follows:(1) patients with pathologically confirmed primary hepatocellular carcinoma prior to receiving any initial antitumor therapy (including surgery, interventional therapy, chemotherapy, or radiotherapy); (2) patients with complete clinical and pathological data, including Edmondson–Steiner histological grading; and (3) patients with no history of previous antitumor treatment before the initial management of HCC. Exclusion criteria were as follows: (1) patients with autoimmune or infectious diseases; (2) patients with significant heart, liver, or kidney dysfunction; (3) patients with other concomitant malignancies; and (4) patients with incomplete or missing clinical data. This study was conducted in accordance with the Declaration of Helsinki and approved by the Ethics Committee of Kaiyuan Langdong Hospital of Guangxi Medical University (2025-Ethics No. 26). All patients provided written informed consent before participating in the study (**Figure 1**).

### 2.2 Data acquisition and preparation

Patients were followed up regularly at the outpatient department of our hospital. The collected clinical data included basic patient information (age, sex, and medical history), pre-treatment routine blood test parameters, pathological examination results, and follow-up information. Recorded indicators included CRP, ALB, absolute counts of neutrophils and lymphocytes, creatinine, total bilirubin (TBIL), INR, maximum tumor diameter, number of tumors, platelet count, AFP, CD3^+^CD4^+^ (helper T lymphocytes), and CD3^+^CD8^+^ (cytotoxic T lymphocytes). The tumor diameter was based on post-treatment pathological findings. Tumor recurrence was assessed based on characteristic features identified in patients’ imaging examinations and laboratory test results.

### 2.3 Pre-treatment inflammation-related and other prognostic scores and their classifications

General clinical data and hematological test results of the patients were collected to calculate pre-treatment prognostic scores related to primary liver cancer. These scores included mGPS, MELD, tumor burden (TBS), aMAP score, ALBI score, PALBI score, CRAFITY score, PLR, PNI, NLR, SII, and CD4/CD8 ratio. Except for TBS, the remaining 11 scores were categorized as follows: PLR = Platelet count/lymphocyte count, Commonly dichotomized at 150–200; PLR ≥200 indicates high inflammation. CD4/CD8 ratio = CD3^+^CD4^+^ T cells/CD3^+^CD8^+^ T cells. SII = (Platelet count × Neutrophil count)/lymphocyte count, SII ≥330–400 ×10^9^/L is typically considered high. NLR = Neutrophil count/lymphocyte count, NLR ≥3.0 or 3.5 indicates elevated systemic inflammation. MELD = 9.57 × ln (creatinine [mg/dL]) + 3.78 × ln (TBIL [mg/dL]) + 11.2 × ln (INR) + 6.43. PNI = ALB (g/L) + 5 × total lymphocyte count (10^9^/L), PNI <45 indicates poor nutritional/immunologic status. aMAP = (0.06 × age) + (0.89 × gender [male = 1, female = 0]) + (0.48 × ALBI score) − (0.01 × platelet count [10^9^/L]) + 7.4, mGPS: CRP ≤ 10 mg/L → 0, CRP > 10 mg/L and ALB ≥ 35 g/L → 1, CRP > 10 mg/L and ALB < 35 g/L → 2. ALBI = (log_10_ TBIL [μmol/L] × 0.66) − (ALB [g/L] × 0.085). Grades: ≤−2.60 (Grade 1); >−2.60 to ≤−1.39 (Grade 2); >−1.39 (Grade 3). PALBI = 2.02 × log_10_ TBIL [μmol/L] − 0.37 × (log_10_ bilirubin)^2^ − 0.04 × ALB [g/L] − 3.48 × log_10_ platelet count [10^9^/L] + 1.01 × (log_10_ platelet)^2,^ Grades: ≤−2.53 (Grade 1); >−2.53 to ≤−2.09 (Grade 2); >−2.09 (Grade 3). CRAFITY: Based on thresholds of CRP and AFP values, usually defined in immunotherapy cohorts (exact criteria vary by study; one common version assigns 0–2 points based on CRP > 1 mg/dL and AFP > 100 ng/mL). Tumor Burden Score (TBS):TBS = √(a² + b²), where a = maximum tumor diameter (cm) and b = number of tumors. Low TBS (≤3.36), medium TBS (>3.36–13.74), and high TBS (>13.74).

### 2.4 Follow-up content and timing (post-hepatectomy/systemic therapy)

Research Electronic Data Capture (REDCap) is a secure, efficient, web-based platform for clinical research data collection and follow-up management, employed in our post-treatment follow-up and prognostic studies of patients with liver cancer. In HCC follow-up, REDCap facilitates structured collection and management of multidimensional data, including patients’ baseline information, pre-treatment examinations, imaging data, laboratory indicators (including AFP, ALBI, NLR, and SII), and survival status. Its built-in logic branching, automated score calculations, and follow-up reminder functions markedly improve follow-up efficiency and data quality. Additionally, it provides robust data export capabilities, facilitating survival analysis and Cox regression modeling. REDCap facilitates multi-user permission settings, audit trail logging, and local server deployment, meeting privacy protection and regulatory compliance requirements for clinical data management. REDCap has become an essential platform for long-term post-treatment follow-up, recurrence monitoring, and multicenter collaborative research in liver cancer. Follow-up content and timing (post-hepatectomy/systemic therapy): For the initial 2 years after surgery, follow-up was every 3 months. From years 2 to 5, follow-up was every 6 months. After 5 years post-surgery, follow-up was every 12 months, up to 10 years in total.

The REDCap system was implemented by requesting and installing the official source code. After verification and corrections, user accounts were established, granting access to the platform for the design project-specific data collection instruments. This entailed establishing projects, designing data collection instruments, and formulating individual data entry fields. For multicenter studies (note: this study did not include multicenter data), a unified research protocol should be developed, with the lead institution responsible for case report form (CRF) design in collaboration with all participating centers. In clinical trials, CRFs in REDCap are designed to restrict the data entry format and field types. Field validation functions are employed to flag missing or out-of-range values, thereby preventing invalid data entries. During CRF completion, REDCap uses color-coded indicators to signify data completeness: red denotes incomplete fields, yellow indicates unverified entries (pending data manager review), and green represents fully completed entries. These visual cues facilitate the project staff’s efficient monitoring and management. Establishing data access groups (DAGs) and adding members from respective centers is a crucial stage in the allocation of user permissions. As an application-based data management system, proper user permission configuration is essential and relates to ethical, reliability, and security concerns. Data administrators possess complete control over the creation, modification, and deletion of DAGs. Conversely, quality control and data entry personnel are restricted to data input, export, and analysis functions, without permission to modify database structures or delete records.

### 2.5 Statistical methods

Statistical analyses were performed using Statistical Package for the Social Sciences software (version 20.0) and RStudio (version 4.1.0). An independent samples t-test was utilized for comparison between two groups, and analysis of variance was utilized for comparison between multiple groups. Kaplan–Meier survival analysis was employed to assess the survival outcome of patients with liver cancer between the two groups, and the log-rank test was applied for statistical comparison between groups. Univariate and multivariate Cox proportional hazards regression analyses were performed to identify prognostic factors. Variables with statistical significance (*P* < 0.05) in the univariate Cox analysis were subsequently included in the multivariate Cox regression to develop the prognostic model. The predicted efficacy of different scoring systems for post-treatment survival among patients with liver cancer was evaluated using the receiver operating characteristic (ROC) curve. *P* < 0.05 was considered statistically significant.

## 3 Results

### 3.1 Baseline data of the patients

This study included 304 patients who underwent liver cancer treatment. In the training set, 88.9% of the patients were males and 11.1% were females, with a median age of 49 years (range: 22–81). The median ALBI score was -2.64 (range: –3.78 to –1.38), and the median PALBI score was –3.56 (range: –4.68 to –1.75). **Table 1** presents the baseline characteristics of the training set. In the validation set, 78.3% of the patients were male and 21.7% were female, with a median age of 49 years (range: 27–71). The median ALBI score was –2.85 (range: –3.68 to –1.49), and the median PALBI score was –3.66 (range: –4.64 to –1.77). **Table 1** presents baseline characteristics of the validation set. Except for Gender (*p* = 0.019), all other clinical characteristics showed no statistically significant differences between the training and validation sets (*p* > 0.05). The proportion of females in the validation set (21.7%) was significantly higher than in the training set (11.1%).

**Table 1:** Baseline characteristics of the included patients.

Feature	The training set (N=189)	The validation set (N=115)	*p* value
Age	49 (22∼81)	49 (27∼71)	0.400
Gender			0.019*
Male	168 (88.9%)	90 (78.3%)	
Female	21 (11.1%)	25 (21.7%)	
AFP (ng/mL)			0.247
<400	103 (54.5%)	54 (47.0%)	
≥400	86 (45.5%)	61 (53.0%)	
Tumor Number			0.633
Single	144 (76.2%)	84 (73.0%)	
Multiple	45 (23.8%)	31 (27.0%)	
BCLC			0.493
0	5 (2.6%)	2 (1.70%)	
A	97 (51.3%)	50 (43.5%)	
B	27 (14.3%)	22 (19.1%)	
C	60 (31.7%)	40 (34.8%)	
mGPS			0.644
0	145 (76.7%)	93 (80.9%)	
1	34 (18.0%)	16 (13.9%)	
2	10 (5.3%)	6 (5.2%)	
CRAFITY			0.855
0	63 (33.3%)	36 (31.3%)	
1	99 (52.4%)	64 (55.7%)	
2	27 (14.3%)	15 (13.0%)	
ALBI	-2.64 (-3.78~-1.38)	-2.85 (-3.68~-1.49)	0.111
PALBI	-3.56 (-4.68~-1.75)	-3.66 (-4.64~-1.77)	0.599

*significantly, Categorical variables (gender, AFP, tumor number, BCLC stage, mGPS, CRAFITY score) were analyzed using the Chi-square test to calculate p-values. Continuous variables (age, ALBI, PALBI) were analyzed using the independent samples t-test (Welch’s t-test) to estimate p-values.

### 3.2 Identification of independent risk factors for post-treatment among patients with HCC

Age, body weight, white blood cell count, prothrombin time, INR, prealbumin, and scoring systems including mGPS, MELD, TBS, aMAP, ALBI, PALBI, CRAFITY, PLR, PNI, NLR, SII, and the CD4/CD8 ratio were included in univariate and multivariate Cox regression analyses. Results from the univariate Cox regression analysis revealed that prealbumin (*P* = 0.01), PNI (*P* < 0.001), TBS (*P* = 0.01), ALBI (*P* < 0.001), PALBI (*P* < 0.001), and CRAFITY (*P* < 0.001) scores were potential risk factors for post-treatment recurrence among patients with HCC. Further multivariate Cox regression analysis identified PALBI (HR: 1.92; 95% CI: 1.05–3.5; *P* = 0.03) and CRAFITY (HR: 1.41; 95% CI: 1.01–1.97; *P* = 0.04) as independent prognostic factors associated with recurrence after liver treatment among patients with HCC. **Table 2** presents detailed results.

**Table 2:** Identification of independent risk factors.

Hazard factor	Univariate analysis		Multivariate analysis	
	HR (95% CI)	P-value	HR (95% CI)	P-value
Age	1.00 (0.98-1.01)	0.76		
Weight	0.99 (0.97-1.01)	0.31		
White Blood Cell	0.99 (0.89-1.09)	0.78		
PT (s)	1.07 (0.94-1.21)	0.31		
INR	1.06 (0.91-1.23)	0.43		
Prealbumin	1.00 (0.99-1.00)	0.01	1.00 (1.00-1.00)	0.41
mGPS	1.25 (0.89-1.76)	0.19		
MELD	1.02 (0.98-1.07)	0.36		
PNI	0.94 (0.91-0.98)	0.00	1.00 (0.94-1.06)	0.95
NLR	1.13 (0.99-1.29)	0.07		
TBS	1.04 (1.01-1.08)	0.01	1.04 (1.00-1.07)	0.05
ALBI	2.48 (1.45-4.25)	0.00	1.19 (0.46-3.06)	0.71
aMAP	1.02 (0.99-1.04)	0.16		
PALBI	2.15 (1.31-3.53)	0.00	1.92 (1.05-3.50)	0.03
CRAFITY	1.57 (1.15-2.13)	0.00	1.41 (1.01-1.97)	0.04
SII	1.00 (1.00-1.00)	0.69		
PLR	1.00 (1.00-1.01)	0.28		
CD4/CD8	0.89 (0.73-1.08)	0.23		

### 3.3 Construction of the prognostic model and nomogram

**Figure 2:**
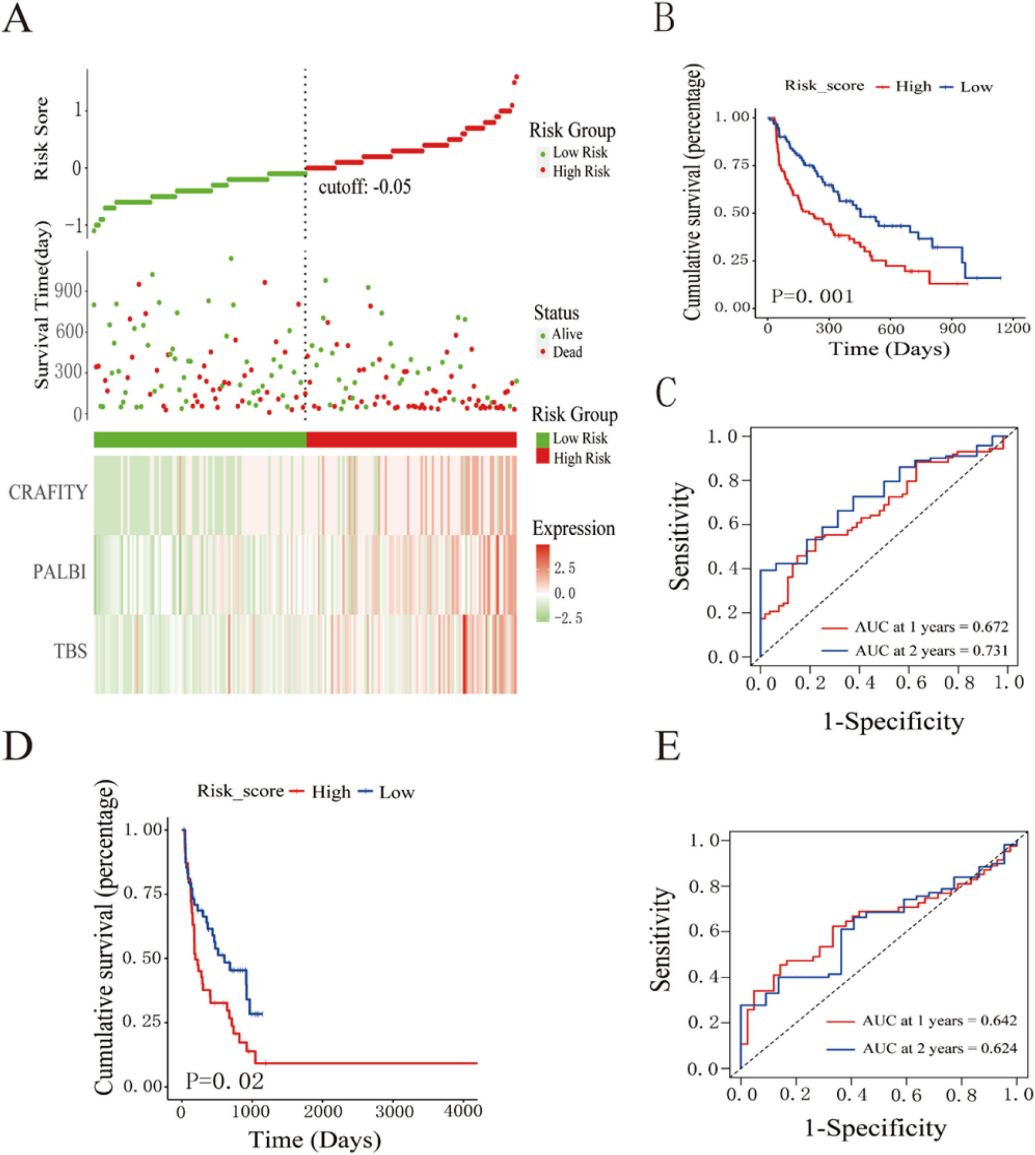
Prognostic model based on TBS, PALBI, and CRAFITY scores. **(A)** Distribution of risk scores, survival status of patients across risk groups, and heatmaps of the three scoring systems. **(B, C)** Kaplan–Meier survival curves and time-dependent ROC curves in the training cohort. **(D, E)** Kaplan–Meier survival curves and time-dependent ROC curves in the validation cohort.

**Figure 3:**
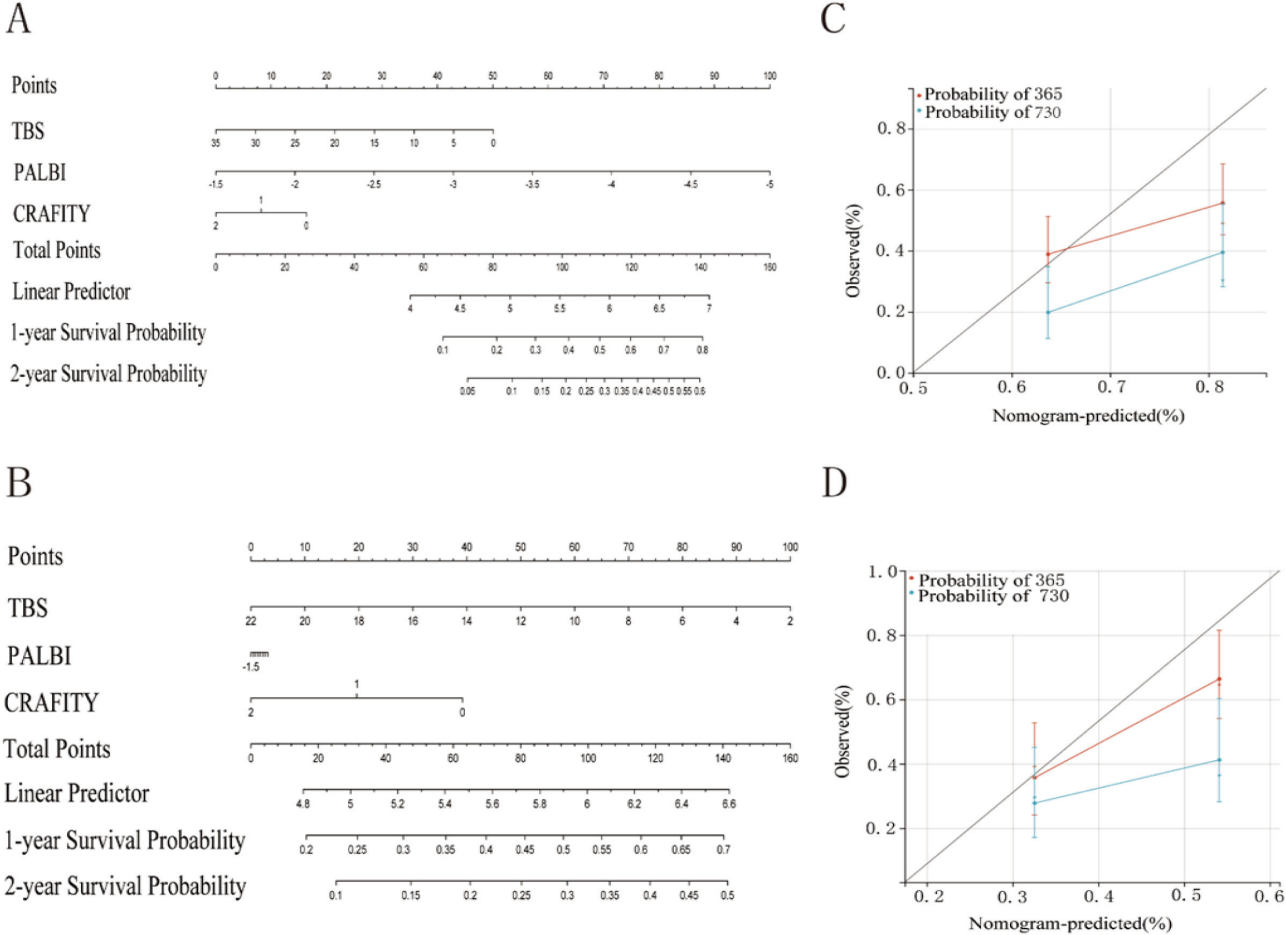
Nomogram for predicting 1- and 2-year overall survival among patients with HCC. **(A)** Nomogram predicting 1- and 2-year survival probabilities in the training cohort. **(B)** Nomogram predicting 1- and 2-year survival probabilities in the validation cohort. **(C, D)** Calibration curves for training **(C)** and validation **(D)** cohorts.

Parameters with statistical significance (*P* < 0.05) in the univariate Cox regression analysis were included in a forward stepwise regression model to perform multivariate Cox analysis and construct a prognostic model. The resulting risk score formula was as follows: Risk score = 0.03903 × TBS + 0.79809 × PALBI + 0.40881 × CRAFITY. Risk scores were calculated for each patient based on this model, and patients were stratified into high- and low-risk groups according to the median risk score (**Figure 2A**). Kaplan–Meier survival analysis revealed that patients in the high-risk group exhibited significantly worse prognosis compared to those in the low-risk group within the training cohort (**Figure 2B**). Time-dependent ROC curve analysis revealed that the model yielded an AUC of 0.672 for predicting 1-year survival and 0.731 for predicting 2-year survival after surgery (**Figure 2C**). Similarly, in the validation cohort, Kaplan–Meier analysis revealed poorer outcomes for the high-risk group (**Figure 2D**). The ROC analysis in the validation set revealed AUC values of 0.642 and 0.624 for predicting 1- and 2-year survival, respectively (**Figure 2E**). Nomograms are commonly utilized as a visual tool for presenting prognostic models. Using the rms package in R, we developed a nomogram to predict 1- and 2-year OS for patients with HCC after surgical treatment, integrating multiple prognostic factors into a single, user-friendly interface. The nomogram demonstrated good predictive ability in training and validation cohorts. The concordance index (C-index) of the model was 0.662 in the training cohort (**Figure 3A**) and 0.611 in the validation cohort (**Figure 3B**). The bootstrap method was utilized with 1,000 resampling iterations to further evaluate the nomogram’s predictive accuracy. Calibration curves indicated good agreement between predicted and observed survival probabilities in training and validation cohorts (**Figures 3C,D**).

### 3.4 Classification of scoring systems and construction of nomogram models

**Figure 4:**
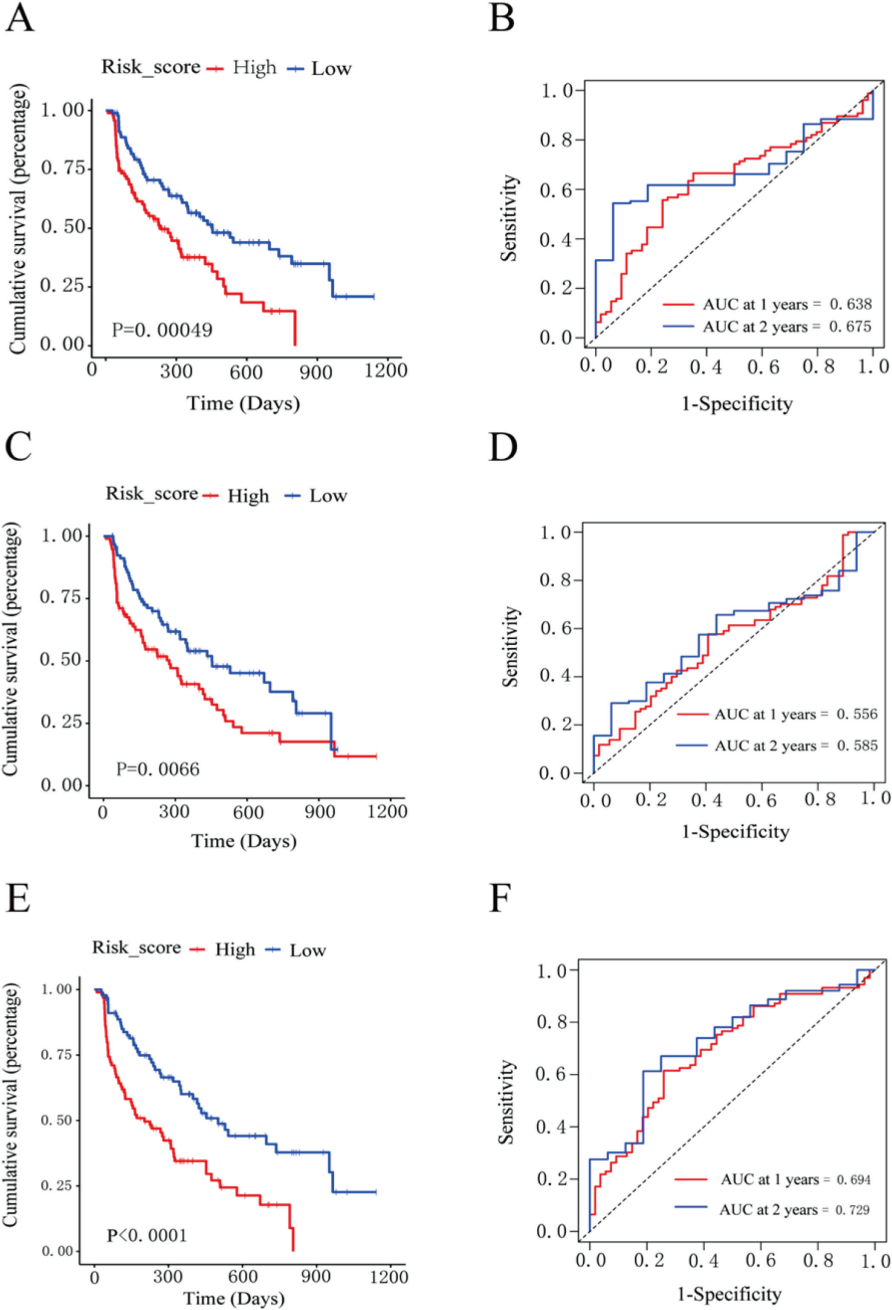
Prognostic models are based on three different categories of scoring systems. **(A, B)** Kaplan–Meier survival curves and time-dependent ROC curves for the training cohort using a prognostic model based on nutrition- and metabolism-related scores. **(C, D)** Kaplan–Meier survival curves and time-dependent ROC curves for the training cohort using a prognostic model based on immune cell-related scores. **(E, F)** Kaplan–Meier survival curves and time-dependent ROC curves for the training cohort using a prognostic model based on inflammation-related scores.

Excluding the TBS score, the remaining 11 scoring systems were classified into three categories: Inflammation-related scores: mGPS, ALBI, PALBI, and CRAFITY. Nutrition and metabolism-related scores: MELD, PNI, and aMAP. Immune cell-related scores: PLR, CD4/CD8 ratio, SII, and NLR. A prognostic model was developed by incorporating MELD, PNI, and aMAP scores into a multivariate Cox regression analysis. The risk score was calculated utilizing the following formula: Risk score = MELD × 0.01947 + PNI × –0.05702 + aMAP × 0.00305. Patients were stratified into high- and low-risk groups based on the median risk score. In the training cohort, patients in the high-risk group exhibited significantly worse prognosis compared to those in the low-risk group (**Figure 4A**). Time-dependent ROC analysis revealed that this model yielded AUCs of 0.638 and 0.675 for predicting 1- and 2-year post-treatment survival, respectively (**Figure 4B**). However, in the validation cohort, no statistically significant difference was observed in survival between high- and low-risk groups, with AUCs of 0.543 for 1-year and 0.573 for 2-year survival. A prognostic model was developed using PLR, CD4/CD8 ratio, SII, and NLR scores through multivariate Cox regression. The risk score formula was: Risk score = PLR × 0.004516112 + CD4/CD8 × –0.131596028 + SII × –0.000967151 + NLR × 0.243616128. Based on the median score, patients were grouped into high- and low-risk subgroups. Kaplan–Meier analysis in the training set revealed that high-risk patients exhibited poorer outcomes (**Figure 4C**). ROC analysis revealed AUCs of 0.556 and 0.585 for 1- and 2-year survival prediction, respectively (**Figure 4D**). In the validation set, no statistically significant difference was observed in survival between the two groups, with AUCs of 0.514 and 0.479, respectively. A prognostic model was developed using mGPS, ALBI, PALBI, and CRAFITY scores. The formula was: Risk score = mGPS × –0.2118755 + ALBI × 0.5590588 + PALBI × 0.4991822 + CRAFITY × 0.517295.

**Figure 5:**
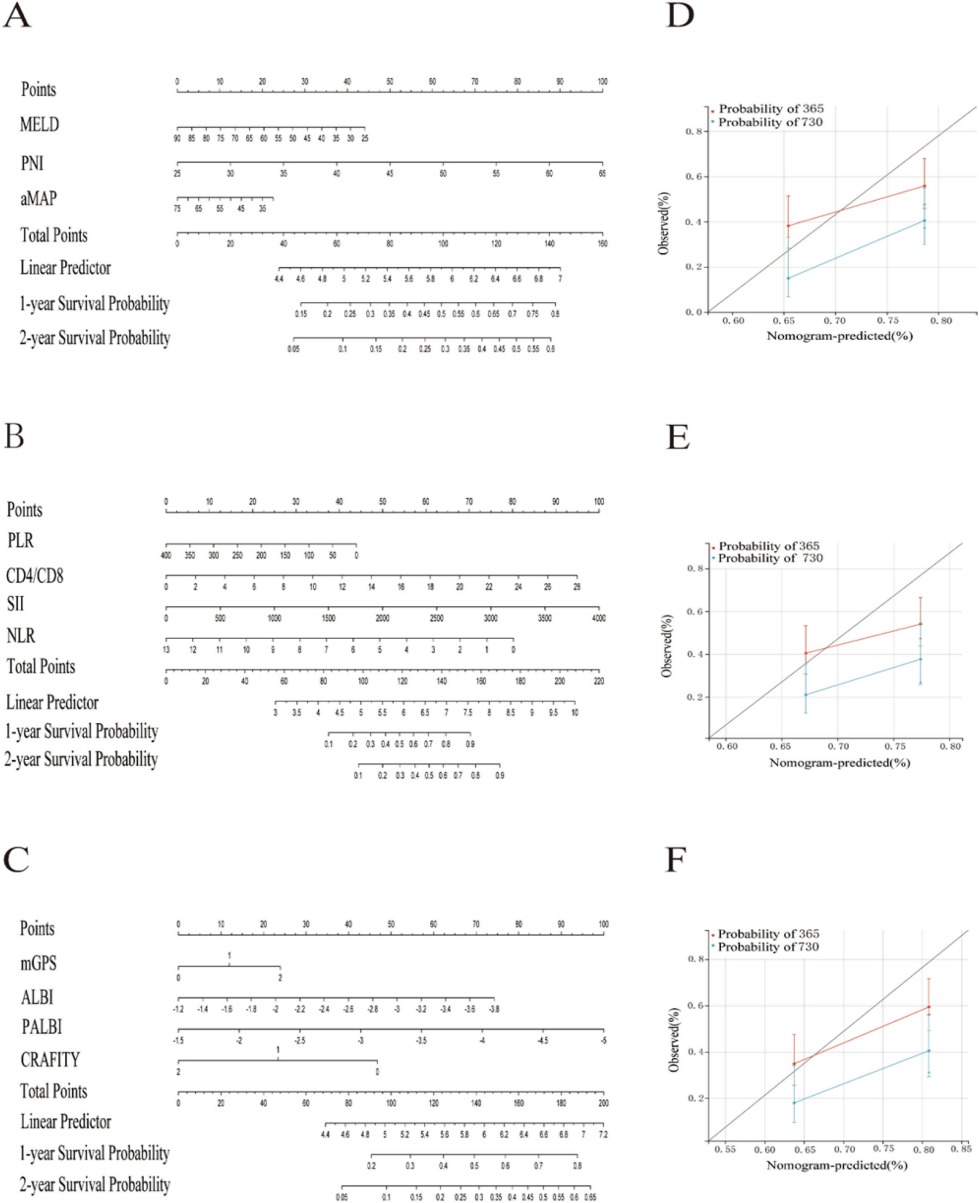
Nomogram models were constructed based on three different types of scoring systems. Nomograms based on the nutrient metabolism-related score **(A)**, immune cell-related score **(B)**, and inflammatory response-related score **(C)** were developed to predict 1- and 2-year survival probabilities of patients with HCC in the training cohort. Calibration curves for the three models **(D–F)** demonstrated good agreement between the predicted and observed 1- and 2-year survival rates in the training cohort.

**Figure 6:**
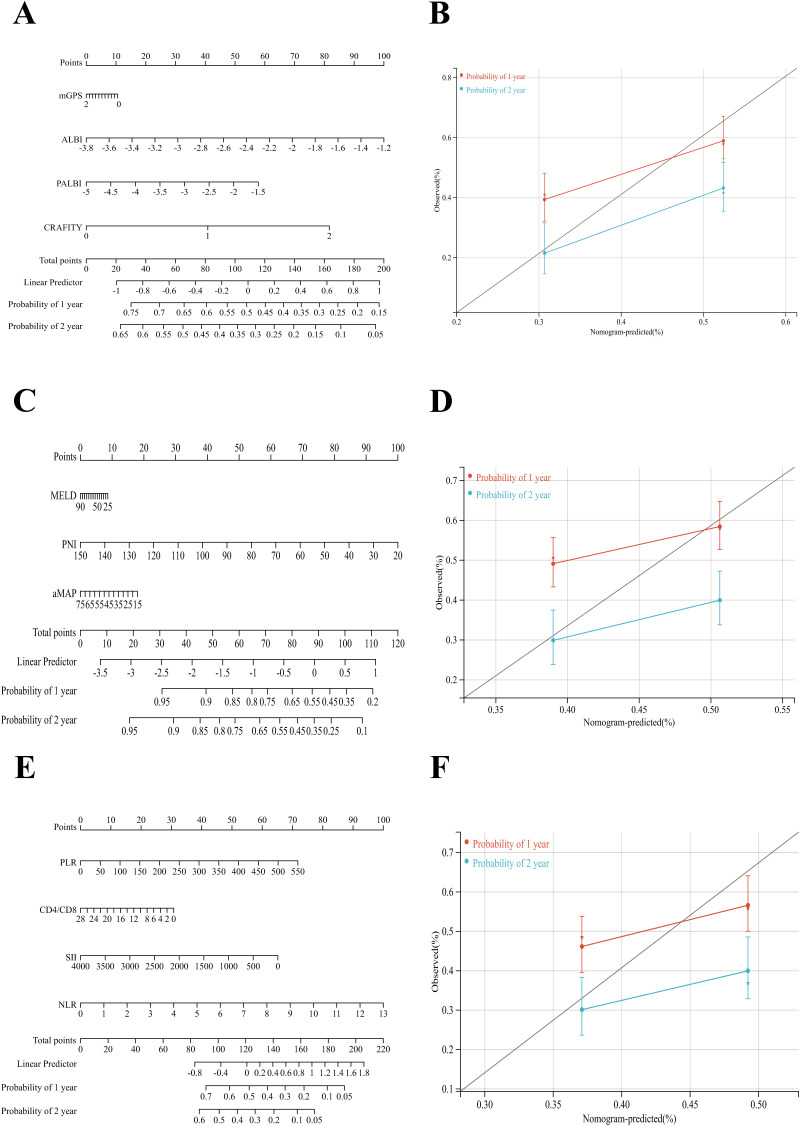
Nomograms were constructed based on three different types of scoring systems within the overall dataset. **(A)** inflammatory response-related score **(B)** metabolism-related score and **(C)** immune cell-related score were developed to predict 1- and 2-year survival probabilities of patients with HCC in the training cohort. Calibration curves for the three models **(D–F)** demonstrated good agreement between the predicted and observed 1- and 2-year survival rates in the training cohort.

Patients were stratified into high- and low-risk groups based on the median score. In the training cohort, high-risk patients exhibited significantly worse survival (**Figure 4E**). This model achieved AUCs of 0.694 and 0.729 for predicting 1- and 2-year post-treatment survival, respectively (**Figure 4F**). In the validation cohort, a significant difference was observed in the prognosis between high- and low-risk groups, with AUCs of 0.631 and 0.640, respectively. Nomogram models based on each of the three scoring categories were developed to predict 1- and 2-year post-treatment survival among patients with HCC (**Figures 5A–C**). Among them, the nomogram based on inflammation-related scores exhibited the best predictive performance in the training set. The overall C-index values were as follows: 0.627 for the nutrition/metabolism-related model, 0.596 for the immune cell-related model, and 0.653 for the inflammation-related model. Calibration curves in the training cohort exhibited good agreement with the ideal reference line (**Figures 5D–F**). However, in the validation cohort, the predictive performance of all three models was relatively poor. The C-index values were as follows: 0.589 for the nutrition/metabolism-related model, 0.564 for the immune cell-related model, and 0.584 for the inflammation-related model. We integrated survival time, status, and four inflammation-related scores to develop a Cox-based nomogram, evaluating their prognostic significance in the total sample. The model achieved a C-index of 0.610 (95% CI: 0.569–0.652; P = 1.63x10^-7,**Figures 6A, B**). Next, a nomogram incorporating survival data and three nutrition/metabolism features was established, yielding a C-index of 0.581 (95% CI: 0.548–0.614; P = 1.32x10^-6, **Figures 6C, D**). Finally, we developed a nomogram using survival data and four immune cell features, which showed a C-index of 0.575 (95% CI: 0.538–0.613; P = 9.36x10^-5, **Figures 6E, F**). These findings are fundamentally consistent with the results from the discovery set.

## 4 Discussion

Despite the utilization of imaging findings, pathological features, including MVI, and biomarkers such as AFP to predict recurrence, their sensitivity and specificity remain suboptimal. Accordingly, accurately predicting HCC recurrence remains a clinical challenge. This study aimed to develop a prognostic model based on multiple HCC-related scoring systems using a carefully designed randomized, retrospective approach to predict post-treatment prognosis among patients, given the accessibility of routine laboratory test results (44, 45).

Univariate and multivariate Cox regression analyses were performed on 12 scoring systems, identifying PALBI and CRAFITY scores as independent prognostic predictors. Subsequently, a prognostic model was constructed based on TBS, PALBI, and CRAFITY scores, and risk scores were calculated for each patient with HCC. Patients were stratified into high- and low-risk groups according to the median risk score. The analysis of post-treatment outcomes in both cohorts revealed strong predictive performance of the model (45).

Tumor-associated inflammatory responses and the TBS reflect tumor morphology and burden (46). Studies have demonstrated that TBS possesses superior discriminatory capability to the Milan criteria and other tumor-specific scores, with each one-point increase in TBS correlating to a 6% increase in mortality risk (47). These findings indicate that TBS is an independent risk factor for PFS and OS and holds substantial prognostic value for patients undergoing hepatic resection (48).

The PALBI score, a recently proposed method for assessing liver function, integrates objective and easily accessible parameters, providing a more accurate assessment of hepatic reserve. Luo Hongmei’s team assessed 785 patients with HBV-related HCC undergoing hepatic resection at West China Hospital and found that higher PALBI grades were associated with larger tumors, increased MVI, portal vein tumor thrombus, poor differentiation, cirrhosis, higher recurrence rates, and lower long-term survival. PALBI was an independent predictor of recurrence-free and OS (49). Wang et al. reported that dynamic PALBI monitoring more accurately presented the impact of liver dysfunction on prognosis, with stable PALBI scores indicating lower recurrence rates and better long-term outcomes (24).

The CRAFITY score is derived from the inflammation marker CRP and the tumor marker AFP (30). Inflammation is essential in tumor initiation and progression, and CRP is a well-recognized acute-phase protein indicative of systemic inflammation in cancer (30). CRP and AFP are commonly utilized laboratory markers in HCC, making CRAFITY an objective and broadly applicable scoring system (30). Previous studies support the utility of the CRAFITY score in predicting outcomes of patients with HCC receiving immunotherapy (50). This study categorized the 12 scoring systems into three types: Nutrition and metabolism-related: MELD, PNI, and aMAP. Immune cell-related: PLR, CD4/CD8 ratio, SII, and NLR. Inflammation-related: mGPS, ALBI, PALBI, and CRAFITY. Prognostic models based on each category were developed, and their predictive performance was evaluated in training and validation cohorts. The model based on inflammation-related scores exhibited the most significant predictive power. Stratification based on median risk score indicated significantly worse outcomes in the high-risk group (*P* < 0.0001). ROC analysis revealed that this model achieved an AUC of 0.694 and 0.729 for predicting 1- and 2-year survival, respectively, outperforming the other two models.

Inflammation is central to HCC pathogenesis (51). Inflammatory cells infiltrating the tumor microenvironment interact with tumor cells through complex pathways (51). Compared with the original model, the inflammation-based model included additional parameters, including mGPS and ALBI (52). The mGPS integrates CRP and serum ALB, serving as a critical indicator of systemic inflammation. The correlation between cancer and inflammation is now well established. CRP denotes infection and disease severity, whereas hypoalbuminemia indicates poor nutritional status and impaired liver function, both of which are prevalent in HCC due to tumor-associated protein metabolism disturbances. mGPS effectively assesses both nutritional and inflammatory status, providing advantages including ease of measurement, low cost, and high prognostic value (53). Inflammation plays a pivotal role in the initiation, progression, and recurrence of HCC (54). Chronic inflammatory conditions (such as hepatitis B or C virus infection, nonalcoholic steatohepatitis, and cirrhosis), induce sustained hepatocyte injury and regeneration, thereby promoting genomic instability and malignant transformation (55). Inflammatory mediators, including interleukin-6 (IL-6), tumor necrosis factor-α (TNF-α), and reactive oxygen species, activate oncogenic signaling pathways NF-κB and STAT3, which enhance tumor cell proliferation, survival, and invasiveness (56). In addition, inflammation profoundly alters the tumor immune microenvironment by facilitating tumor-associated macrophage polarization and impairing cytotoxic T-cell activity, ultimately promoting immune evasion (57). Following curative resection or locoregional therapy, residual microscopic lesions are more likely to recur under such a pro-inflammatory milieu (58). Therefore, modulating systemic and local inflammation represents a promising strategy to suppress HCC recurrence and improve long-term outcomes (54).

Sokolov et al. reported that high mGPS scores were significantly associated with shorter cancer-specific survival (59). The pre-treatment evaluation of mGPS aids in identifying subgroups of patients with locally advanced colorectal cancer who may benefit from more aggressive surgery (59). Similarly, pre-treatment liver reserve is a critical factor for long-term prognosis among patients with HCC (59). The ALBI grade has been proposed as a tool to evaluate hepatic functional reserve (60). ALBI grades 2–3 correlate with advanced tumor stage, vascular invasion, and poor prognosis in HCC (61). Therefore, ALBI grading functions as a promising prognostic marker for patients undergoing curative resection (61).

Many patients with HCC have underlying chronic liver disease, which affects common blood parameters and may offer prognostic value. Tumor progression is closely associated with inflammation and immune dysregulation, which manifests as altered inflammatory and immune biomarkers in routine blood tests (62, 63). The inflammation-related scores discussed above are derived from standard laboratory data without necessitating additional testing. These parameters are simple, inexpensive, readily available, and objective, minimizing operator variability. Consequently, their role in the prognostic assessment of HCC should be further studied in a systematic and detailed manner.

This study has some limitations. First, its retrospective design and inclusion of only Chinese patients, primarily with a relatively small sample size and a predominance of HBV- or HCV-related cases, may limit generalizability. Although the prognostic value of inflammation-based indicators is increasingly recognized, most supporting studies are retrospective and subject to publication bias against negative results. Additionally, inconsistent cutoff values hinder cross-study comparison and clinical implementation. Prospective studies are urgently needed to validate the prognostic utility of these inflammatory markers and to investigate the underlying molecular mechanisms. This could lead to the identification of optimal indicators and enable mechanism-based, personalized interventions to improve post-treatment outcomes in HCC. Meanwhile, we recognized the potential overfitting in our study. This discrepancy may be attributed to the limited sample size and the single-center nature of the dataset. In future research, we plan to conduct a prospective, multi-center study to further validate and refine our model, Although our study was conducted in a medical oncology department, future work will include validation in HCV-related and surgical cohorts.

## 5 Conclusions

Pre-treatment PALBI and CRAFITY scores emerged as independent prognostic factors for primary HCC. A prognostic model constructed using TBS, PALBI, and CRAFITY demonstrated predictive power for assessing post-treatment prognosis among patients. Among various scoring systems, inflammation-related scores (mGPS, ALBI, PALBI, and CRAFITY) exhibited a closer association with post-treatment outcomes than metabolism-related (MELD, PNI, and aMAP) and immune-related scores (PLR, CD4/CD8, SII, and NLR). These findings emphasize the pivotal role of systemic inflammation in HCC progression and recurrence and support the clinical use of inflammation-based scores for objective risk stratification. Future large-scale, multicenter prospective studies are needed to validate these models and investigate the integration of molecular biomarkers and dynamic score monitoring to further enhance individualized post-treatment surveillance and treatment strategies in HCC.

## Data Availability

The raw data supporting the conclusions of this article will be made available by the authors, without undue reservation.
